# Insulin dose adjustment policy for certified diabetes care and education specialists: Safe and effective

**DOI:** 10.1016/j.jcte.2025.100423

**Published:** 2025-11-01

**Authors:** Jeannine C. Leverenz, Lauren Horton, Barry Conrad, Shannon Lin, Annette Chmielewski, Franziska K. Bishop, Melissa Quaid, Priya Prahalad, David M. Maahs

**Affiliations:** a Lucile Packard Children’s Hospital, Division of Pediatric Endocrinology, Palo Alto, CA, United States; b Division of Endocrinology, Department of Pediatrics, School of Medicine, Stanford University, Stanford, CA, United States; c Stanford Diabetes Research Center, Stanford, CA, United States

**Keywords:** Type 1 diabetes, Continuous glucose monitoring, Remote patient monitoring, Diabetes technology, Glycemic maintenance, Time in range, Insulin dosing

## Abstract

In 4T Study 1, youth with new-onset type 1 diabetes started a continuous glucose monitor (CGM) soon after diagnosis and received remote CGM data review and dose changes by a Certified Diabetes Care and Education Specialist (CDCES) via secure portal messaging. We describe the CDCES policy to make incremental dose adjustments and report its safety and effectiveness, which facilitated patients’ reaching and maintaining targets. We aim to publish this data-supported CDCES protocol to facilitate use at other diabetes centers who may restrict CDCES from adjusting insulin doses.

The CDCESs and Pediatric Endocrinologists agreed on criteria for making dose changes. CDCESs made insulin dose adjustments and consulted with Pediatric Endocrinologists per protocol and as needed. CDCES sent messages with suggested dose adjustments and behavior changes via secure portal messaging.

In the first year, a total of 1564 remote patient monitoring messages were sent to 133 participants. Most messages were triggered by low time-in-range (TIR, 70–180 mg/dl [63 %]), hypoglycemia (39 %), decline in TIR (13 %), or insufficient CGM wear time (7 %). There were 3 episodes of severe hypoglycemia, none adjudicated related to the CDCES dosing protocol. At one year, the mean time <70 mg/dl was <2 %, and the A1C target of <7 % was met by 64 %.

We created a policy for CDCESs to adjust insulin doses and increase patient interaction between visits. The results demonstrate that CDCES can work at the top of their certification to adjust insulin doses to achieve target goals without decreasing safety.

## Highlights

• Youth with Type 1 Diabetes (T1D) started CGM soon after diagnosis.
• Certified Diabetes Care and Education Specialists (CDCESs) reviewed CGM data.
• A CDCES-led insulin adjustment protocol was implemented with Endocrinologist support.
• At one year, mean time <70 mg/dL was <2% and 64% achieved A1c <7%.
• The CDCES lead protocol proved safe and effective for insulin dosing.

## Introduction

The Diabetes Control and Complications Trial (DCCT) established the benefits of intensive diabetes management to prevent vascular complications in people with type 1 diabetes (T1DM) [1]. Despite the data from the DCCT and advances in care, a majority of youth with T1DM do not meet glycemic targets [2]. One aspect of the DCCT intervention was frequent insulin dose adjustments by a diabetes care team member. Although this was a landmark clinical trial, translating its findings into clinical practice has been challenging because of clinician time constraints. Furthermore, despite the increased use of continuous glucose monitors (CGM), there are barriers to implementing glucose data-sharing technology to facilitate insulin dose adjustment between the recommended quarterly clinical appointments.

To improve outcomes in youth with T1DM, the Stanford Medicine Children’s Health (SMCH) Diabetes team implemented the 4T (Teamwork, Targets, Technology, and Tight Glycemia) program to enhance early diabetes management. The 4T study team published findings on glycemic outcomes [3,4], patient perspectives on early CGM initiation[5], the impact of uninterrupted CGM access on A1C [6], population-level strategies of T1DM [7,8], technology-enabled care models [8,9], the financial sustainability of remote patient monitoring (RPM) [10] and RPM billing [11], and approaches to weight management and physical activity in T1DM [12]. The team also outlined future directions [13] and highlighted the essential role of the Certified Diabetes Care and Education Specialist (CDCES) in developing and sustaining the program [14].

A CDCES is a member of the healthcare team (including on our team Registered Nurses (RN) and Registered Dieticians (RD)) with extensive additional training in diabetes (1000 h) [15] who has been certified by the Certification Board for Diabetes Care and Education. SMCH Division of Pediatric Endocrinology empowers the CDCES to work at the top of their certification within a clearly defined protocol that includes Pediatric Endocrinologist consultation as needed. A goal of the CDCES is to encourage and support the person and family with diabetes through Diabetes Self-Management, Education and Support/Training (DSMES/T) to make their own dose adjustments and become more independent in their diabetes management. To achieve the goal of ensuring the 4T program is sustainable and scalable, we drew upon the strengths and skills of the CDCES team and prioritized top-of-certification tasks. According to the scope of practice, standards of practice, and standards of professional performance for CDCESs, one of the top of certification tasks includes the adjustment of existing treatment regimens, including insulin and oral medications [[16], [17], [18]]. Despite these qualifications and national training certifications, not all hospitals empower CDCES to make insulin dose adjustments.

In the 4T program, youth with new-onset T1DM were started on CGM in the first month of diagnosis and received weekly remote CGM data review by a CDCES, with dose changes and patient education shared via secure portal messaging. Each CDCES had their own list of patients in TIDE (Timely Interventions for Diabetes Excellence), a tool developed with our Stanford Engineering collaborators that gathers data from Dexcom Clarity to facilitate and expedite CGM data review. The TIDE tool defines the patients not meeting targets so the CDCES knows who to prioritize when reviewing data [8,19].

Youth enrolled in 4T Study 1 (2020–2022) had a 1.1 % improvement in A1C and an increase from 28 % to 64 % meeting an A1C < 7 % at one year compared with historic control subjects [4]. The CDCES team has been involved in the 4 T study development and implementation at the SMCH Diabetes Clinic since its inception. The CDCES team adjusts insulin doses based on RPM CGM data. An integral part of the process was development of an insulin adjustment protocol for the CDCES to make insulin adjustments within the scope of their certification and training.

Our objective in this article is first to report the safety and effectiveness of utilizing CDCESs to make incremental dose adjustments between clinic visits with the Pediatric Endocrinologist or Nurse Practitioner. Second, we describe the policy the CDCES team used to make dose adjustments and its current format to share with other diabetes teams. Our protocol approach aims to increase patient contact and to support patients in achieving and maintaining target goals, thereby demonstrating the safety and effectiveness of CDCES working at the top of their certification.

## Methods

Participants in the 4T study provided informed consent and assent to participate. The Stanford University Institutional Review Board approved the study (clinicaltrials.gov: NCT03968055, NCT04336969). The 4T Study 1 included CGM initiation within 30 days of T1DM diagnosis, followed by weekly RPM and patient-reported outcome surveys. The 4T study employed a systemic approach, offering all newly diagnosed youth the program regardless of the language spoken, insurance status, or provider referral. Participants without insurance or with delayed insurance coverage for CGM were provided with devices for up to one year, and those lacking a compatible smartphone received an iPod Touch to enable data sharing for RPM. Care was delivered in the preferred language of each participant or their caregivers. All participants used CGM.

The 4T Study 1 study design and results have been previously published [4]. Briefly, 159 youths with T1DM were assessed for eligibility, and 133 were enrolled in the study (84 %). Among those who did not participate, reasons included a diabetes diagnosis other than T1DM (n = 7), receiving care outside of SMCH (n = 2), declining consent for CGM data integration for RPM (n = 13), and enrolling in another research study (n = 9). Reasons for exclusion were not mutually exclusive. The CDCES team developed a policy within the Division of Pediatric Endocrinology and Diabetes and our hospital to adjust insulin doses by up to 10–20 % or 1 unit (Table 1).

**Table 1:** Dosing policy details – safe and appropriate non-urgent insulin dose adjustments made by CDCES team using glucose trends.

Problem	Considerations	Recommended changes
Glucose results low (>4% Low or > 1 % Urgent Low)	*URGENT: patient having >4% low glucose or > 1 % glucose < 55 mg/dl over the past week of glucose data and parents have not read patient portal message in past 3 days	Contact family by phone
	Lows are occurring at night	Decrease long-acting insulin by 10–20 % unless in honeymoon phase, then may need to decrease by up to 50 % −OR− Adjust basal insulin by 10–20 % unless in honeymoon phase, then may need to decrease by up to 50 %
	Post prandial lows	Adjust carb ratio by 10–20 % −OR− Adjust correction factor by 10–20 %
	Glucose lows following glucose highs	Loosen correction factor by 10–20 %
	Exercise patterns	Educate patient/family about the use of 10-15gm uncovered carbohydrates snack prior to exercise −OR− Counsel patient to place pump in exercise mode 1 h before the start of exercise
	Treatment of low glucose	Educate patient/family to decrease the amount of fast-acting carbohydrates being used to treat the episodes of low glucose −OR− Accept patient sleeping with glucose in high 60 s
Decreased Time in Range (TIR) > 15 %	Change in activity level	If patient has become more active, decrease insulin by 10–20 %. If patient has become less active, increase insulin by 10–20 %.
	Puberty	Tighten carbs and correction by 10–20 % −OR− Increase long-acting insulin by 10–20 %
Overall TIR < 70 %	Timing of insulin doses	Always dose insulin before meals. If hyperglycemia occurs during meals, try dosing insulin 10–15 min prior to meal.
	High glucose following meals	Tighten carb ratio by 10–20 %
	High glucose all day long	Tighten correction factor by 10–20 % −OR− Increase long-acting insulin by 10–20 % −AND- Consider giving correction every 3 h
	Receiving insulin via AID	Dose insulin for all carbs consumed
	Receiving insulin via MDI	Dose insulin for each meal and bedtime correction if last meal was 3 h or more ago
Missing Data	Struggling to keep sensors intact	Educate family on techniques to keep sensors intact

Glucose targets for youth with T1DM during the study period was defined as: A1C <7 %, 70 % time in range (TIR, 70–180 mg/dl) with <4 % <70 mg/dl lows and <1 % urgent lows <55 mg/dl to achieve a A1C of <7 %.

## The CDCES RPM team first developed consistent shared definitions of insulin dosing categories and prioritization of CGM metrics

• Basal insulin: long-acting insulin administered 1–2 times/day or rapid-acting insulin administered via insulin pump/automated insulin delivery (AID) in small increments all day long.
• Bolus insulin: rapid-acting acting insulin administered via injections or insulin pump/AID for meals and glucose corrections.
• CGM glucose pattern management: By using the ambulatory glucose profile (AGP), a pattern at a consistent time of day where the CGM glucose trends are outside the target range may be identified. A minimum of 3–7 days of CGM glucose data are to be used to determine patterns.
• CGM Metric Alerts: >1% urgent lows, >4% lows; not meeting goal of 70 % TIR, CGM wear time <50 %.

## The CDCES RPM team then listed data to be considered when making dose changes

• Age and Date of Birth of the patient
• Date of T1DM diagnosis
• Current insulin doses
• Last and next appointment with a provider

## The CDCES team next itemized factors that need to be considered before making dose changes

• Illness
• Potential exercise related to the CGM glucose profile
• Menstruation cycle status
• Honeymoon phase
• Timing of insulin delivery related to meals and snacks
• Adherence to the insulin regime (missed boluses for meals and snacks)
• Health literacy (to guide effective communication with family)

Once the team agreed on the above definitions, and the criteria to consider, it was agreed that the team would prioritize CGM wear time and hypoglycemia before youth not meeting clinical goals of TIR.

### Secure portal messages

Messages were sent securely via the electronic health record (Epic) patient portal to youth/families weekly for the first year with many families opting to continue receiving messages monthly following their first year after diagnosis. The messages include TIR, percent hypoglycemia, education as well as dose adjustments to assist the youth with T1DM to reach the target goals.

### Ascertainment of severe hypoglycemia and diabetic ketoacidosis

There were three episodes of severe hypoglycemia, which, in the context of newly diagnosed youth with T1DM, were not unexpected or related to the study as determined by the study’s data safety monitoring board (DSMB). Adverse events (AE) and serious adverse events (SAE) followed NIH reporting criteria and were monitored systematically. Any AE and SAE were coded according to a systematic rubric (type of occurrence, description of event, study relatedness, and status) and reviewed by the study investigators. Any hypoglycemic events were categorized by level according to the ADA Standards of Care [20]. The DSMB monitored the outcomes of AEs, and outcome information was entered into a log for inclusion in reports to the participating IRBs and NIH as required. The DSMB reviewed any AE to determine if the AE was related to the research project and its severity.

## Results

A total of 1564 RPM messages triggered by 1901 CGM glucose metric alerts (average 11.8 messages per participant) were sent, with most messages triggered by low TIR (63 %), hypoglycemia (39 %), decline in TIR (13 %) or insufficient CGM wear time (7 %). The full cohort characteristics are in Table 2. Some messages were due to multiple metric alerts. Some messages suggested dose adjustments and some were to educate the patient and family or ask them to make behavior changes (i.e.: pre-bolus, give a correction before bed) and many combined these objectives.

**Table 2:** Characteristics of 4T Study 1.

Characteristic	4 T Study 1
n	133
Baseline characteristics	
Age (years) at T1D diagnosis, median (Q1, Q3)	11 (6, 14)
Sex, n (%)	
Male	74 (55.6)
Female	59 (44.4)
Race/ethnicity, n (%)	
Non-Hispanic White	52 (39.1)
Non-Hispanic Black	1 (0.8)
Hispanic	49 (36.8)
Asian or Pacific Islander	11 (8.3)
Other	17 (12.8)
Unknown / Declined to state	3 (2.3)
DKA at diagnosis, n (%)	72 (54.1)
HbA1c (%) at diagnosis, mean (SD)	12.2 (2.4)
Insurance type, n (%)	
Private	83 (62.4)
Public	47 (35.3)
Both	2 (1.5)
No Insurance	1 (0.8)
Primary language, n (%)	
English	112 (84.2)
Non-English	21 (15.8)
Follow-up characteristics	
CGM initiation within 1 year, n (%)	133 (100)
Initiated CGM <= 30 days, n (%)	131 (98.5)
Days to CGM initiation, median (Q1, Q3)	10 (6, 18)
CGM wear time* (%), median (Q1, Q3)	96.4 (89.3, 97.9)
Insulin pump use within 1 year, n (%)	66 (49.6)
Predictive Low-Glucose Suspend	2 (1.5)
Open loop	34 (25.6)
Hybrid closed loop	33 (24.8)
None	67 (50.4)
Days to pump initiation, median (Q1, Q3)	162 (86, 255)

*Percentage of time CGM is worn out of eligible hours of device wear.

There were three episodes of severe hypoglycemia, which were adjudicated as not study-related. One episode was due to a 15-year-old patient who had been diagnosed by an Adult Neurologist with diabetic neuropathy in the first month after diagnosis and instructed to achieve near normal glycemia without discussion with the diabetes care team. This patient subsequently reported fear of hyperglycemia based on the misdiagnosis of diabetic neuropathy and gave repeated boluses, resulting in overnight severe hypoglycemia; the patient was given glucagon nasal spray by his guardian and taken to the emergency department (ED). The clinical care team, patient, and guardian discussed his hyperglycemia fears and made dose adjustments combined with more education. His diagnosis of neuropathy was subsequently changed as he had a back injury, causing the neuropathy. The second patient, a 13-year-old, gave a full dose of insulin for school lunch but only consumed a portion of the meal and then proceeded to vigorous activity in gym class. Glucagon nasal spray was administered, followed by juice. The clinical care team reviewed with the patient and guardian how to avoid future episodes of severe hypoglycemia and appropriate treatment. Lastly, one patient event was due to a 12-year-old patient and family miscalculating the insulin dose and then walking in the mall. This was a lot of activity for a usually inactive child, resulting in a rapid drop in her blood glucose, which led to a seizure. The CGM read 75, honey was given, and emergency medical services were called; no glucagon was administered, and the patient was taken to the ED. In all cases, the clinical care team was in close contact, and dose adjustments, when clinically indicated in addition to diabetes education, were provided. None of the events were related to recent dose adjustment changes by the CDCES based on the review of the RPM messages.

By 12 months post-diagnosis, 64 % of participants in 4T Study 1 met the A1C target of <7 %, whereas 28 % in the historical cohort reached this target.

## Discussion

In the most recent competencies published by the Association of Diabetes Care and Education Specialists in 2020, under the domain of monitoring, it states that a CDCES can interpret the data from glucose monitoring tools and translate the data findings into actionable recommendations based on the plan of care [16]. We report data from our Pediatric CDCES insulin dosing protocol demonstrating its safety and effectiveness. The CDCES team used their competencies established with CDCES certification to help families interpret the data from the CGM and to recommend insulin dose changes along with behavior changes and diabetes education to assist the youth in meeting glycemic targets. Our diabetes team’s short-term goal—especially for the CDCESs—is to help guardians learn how to adjust insulin doses to meet glycemic targets. Long-term, we aim to support newly diagnosed youth with T1DM in becoming confident with self-management before they transition to an adult provider.

The diabetes team at SMCH created a policy for CDCES to safely make insulin dose adjustments that improved glycemic outcomes as published in the 4T Study 1 [4]. Implementing the CDCES insulin dosing policy led to increased communication with families in between regularly scheduled clinic visits and a higher proportion of youth with diabetes achieving target glycemic goals with limited hypoglycemia [4].

We have previously reported patient-reported outcomes, which indicated that increased communication between clinic visits was perceived as supportive by families [5,21,22]. The CDCES team tailored their outreach with thoughtful language and explanations to meet each family’s unique needs [22].

One motivation for this publication is that we are aware that some institutions do not allow CDCESs to make insulin dose adjustments despite this being within their scope of practice. It is standard practice for CDCES to teach youth with diabetes and their family/caregivers to safely manage and adjust their own insulin doses. We assert that these data provide strong support for CDCESs to safely make dose adjustments following a standardized protocol that can be replicated in other Pediatric diabetes clinics. Indeed, within the context of the 4T Study in which CGM glucose is monitored remotely in between clinic visits, this protocol provides increased touch-points between the diabetes team and families in the first year after diagnosis, when families are most eager for diabetes education and when frequent dose adjustments are required. More recently, we have transitioned the 4T Study findings from research to be the standard of care in our clinic. Sustainability of this intensive management will rely on developing business models that provide reimbursement for the additional work that the CDCES provide, which contributes to the improved outcomes. Results of a pilot study to bill for this additional work have been published [11]. Collaboration with hospital systems will be required so that sufficient CDCESs are hired to provide the 4T model care, translate and sustain improved outcomes to routine care, and have a neutral or positive business model. Insufficient staffing of diabetes care teams is a common theme across pediatric diabetes clinics in the US.

Replicating the DCCT outcomes from intensive management provided by the diabetes care team has been a significant challenge for pediatric diabetes clinics in the USA. As demonstrated by the results to date of the 4T program, the CDCES can work at the top of their certification, following a policy to make insulin dose adjustments without decreasing safety (increasing hypoglycemia), thus assisting the person with diabetes in reaching and maintaining target goals. The protocol presented can be applied at other pediatric diabetes clinics to improve outcomes.

## Financial support and Sponsorship

This work was supported in part by the 10.13039/100000002National Institutes of Health P30DK116074 via the Stanford Diabetes Research Center and R18DK122422 to D.M.M. The study is also supported by the National Science Foundation (2205084). CGM supplies for the first month (transmitter, 3 sensors, and receiver per patient) were donated by Dexcom. Funding for iOS devices and some CGM supplies was provided by a grant through the Lucile Packard Children’s Hospital Auxiliaries Endowment.

## Credit authorship contribution statement

**Jeannine C. Leverenz:** Writing – review & editing, Writing – original draft, Methodology, Investigation, Conceptualization. **Lauren Horton:** Writing – review & editing, Methodology. **Barry Conrad:** Writing – review & editing, Methodology, Investigation, Conceptualization. **Shannon Lin:** Writing – review & editing. **Annette Chmielewski:** Writing – review & editing, Methodology, Investigation, Conceptualization. **Franziska K. Bishop:** Writing – review & editing, Writing – original draft, Project administration, Data curation. **Melissa Quaid:** Writing – review & editing. **Priya Prahalad:** Writing – review & editing, Writing – original draft, Methodology, Investigation, Conceptualization. **David M. Maahs:** Writing – review & editing, Writing – original draft, Methodology, Funding acquisition, Conceptualization.

## Declaration of competing interest

The authors declare the following financial interests/personal relationships which may be considered as potential competing interests: D.M.M. and P.P. have received support from Stanford MCHRI, Stanford HAI and the NSF. D.M.M. has had research support from the NIH and his institution has had research support from DexCom Inc.; and has consulted for Abbott, the Leona M. and Harry B. Helmsley Charitable Trust, Lifescan, Sanofi, Medtronic, Provention Bio, Kriya, and Bayer. All other authors declare that they have no competing interests.
